# Clinical significance of quantitative and qualitative detection of BK and JC virus in blood and urine of renal transplantation recipients

**DOI:** 10.12669/pjms.322.8978

**Published:** 2016

**Authors:** Liangwei Qiao, Qingshan Qu, Xin Jiang

**Affiliations:** 1 Liangwei Qiao, Department of Kidney Transplantation, People’s Hospital of Zhengzhou, Zhengzhou, Henan, 450000, China; 2 Qingshan Qu, Department of Kidney Transplantation, People’s Hospital of Zhengzhou, Zhengzhou, Henan, 450000, China; 3 Xin Jiang, Department of Kidney Transplantation, People’s Hospital of Zhengzhou, Zhengzhou, Henan, 450000, China

**Keywords:** BK virus, JC virus, Polyomavirus, Kidney transplant

## Abstract

**Objective:**

To evaluate value of quantitative and qualitative detection of BK virus (BKV) and JC virus (JCV) in timely diagnosing polyomavirus-associated nephropathy (PVAN) occurring inrenal transplantation recipients.

**Methods:**

We collected 306 cases of urine specimen and 310 cases of blood specimen from 306 patients who underwent renal transplant. Levels of BKV and JCV in blood and urine were detected using real-time quantitative polymerase chain reaction (PCR).

**Results:**

Detection rate of BKV DNA was 33.3% (102/306) in urine and 34.8% (108/310); while that of JCV DNA was 30.7% (94/306) and 33.5% (104/310) respectively. The lowest detectable limit of BCK and JCV detection for patients who underwent renal transplant was 2×10^3^ copies/ml, suggesting high specificity and sensitivity.

**Conclusion:**

Real-time quantitative PCR is able to monitor BCV and JCV in renal transplant recipients in a convenient and rapid way, thus it is beneficial for early discovery, diagnosis and treatment of PVAN.

## INTRODUCTION

Polyomavirus belongs to Papovaviridae, among which, BC virus (BCV) and JC virus (JCV) can induce diseases.^1^, ^2^ It has been reported that,^3^ patients who undergo renal transplant usually have weak immune function, which improves possibility of polyomavirus infection. Polyomavirus can be activated again in about 10% ~ 60% renal transplant recipients, but without affecting renal function; 1%~10% renal transplant recipients may have polyomavirus-associated nephropathy (PVAN). 75% sequence of BKV and JCV is homologous, and both of them are the leading cause for PVAN.^4^ It has been found that, 3% or so patients who infect JCV would develop PVAN; but once JCV and BKV are infected by people at the same time, then the incidence of PCVN is 15%.^5^ To date, there is no antiviral drug which is effective in treating PVAN.^6^ The general method is to adjust category and dosage of immunosuppressor to reduce replication of virus; however, this method increases risk of rejection reaction. Thus it is of great clinical significance to make an early diagnosis for PVAN with a rapid and effective detection method.

Currently, the commonly used detection method includes detection of decoy cells in urine at cytological level,^7^ detection of DNA in urine and blood with polymerase chain reaction (PCR) and immunohistochemical examination. People advocate non-invasive detection method gradually, i.e., detecting virus DNA in humor with PCR.^8^ Detecting BKV and JCV with PCR is of high sensitivity and specificity and is helpful in timely discovering and treating polyomavirus infection. On this account, we used real-time quantitative PCR (probe method) to detect content of BCV and JCV in renal transplant recipients, aiming to provide a valuable basis for clinical diagnosis and therapeutic schedule.

## METHODS

Patients who underwent renal transplant in Zhengzhou People’s Hospital, Henan, China from April 2012 to April 2014 were selected as research subjects. All patients included were those who received allograft renal transplantation for the first time, aged over 18 years, adopted immunosuppressive regimen based on calcineurin inhibitor, antiproliferative agents and hormone, and were willing to participate in the study. Patients who suffered from graft failure, failed to be follow up and had participated in other clinical tests were excluded. In all 306 patients were included (male : female: 184 : 122), with an average age of 37.26 ± 2.16 years.

### Experimental specimen and reagent

Urine and blood were collected after renal transplant was over. Blood specimen was collected as follows. First, 3 ~ 5 ml venous blood was extracted from bend of the arm or the back of the hand with sterile syringe and then injected into sterile collection tube loaded with Ethylene Diamine Tetraacetic Acid (EDTA). Plasma was isolated by putting the tubes at room temperature for no more than four hour or centrifuging the blood for 5 minutes at 1600 rpm. The plasma obtained was transferred into 1.5 ml sterile centrifuge tube. Urine (10 ~ 20 ml) was collected from midstream urine in the monitoring. Plasma and urine specimen were both preserved at - 80 °C.

### Method

### Extraction of DNA template

First, 100 μl blood was poured into 1.5 ml centrifuge tube and then added with 50 μl concentrated solution. After being shaken up for 15 s, the tube was centrifuged in a table centrifuge at 13,000 r/min for 10 minutes. 150 μl supernate was absorbed from the upper layer and abandoned. Afterwards, 25 μl lysis solutions were added into centrifuge tube. Sediment left in the centrifuge tube was removed out with sucker and then was blew and washed for five times. After the sediment was thoroughly scattered through 15 shaking, it was incubated at 100 °C. Ten minutes later, it was centrifuged at 13,000 r/minutes for 10 minutes. The supernate obtained, i.e., purified DNA solution, was transferred to new centrifuge tube. Urine loaded in tube was first shaken for 15 s. Then 1 to 1.5 ml urine was put into centrifuge tube and centrifuged at 13, 000 r/min for 10 min. After the supernate was removed, 50 ml lysis solution was added. Then sediment left was removed out from the centrifuge tube with sucker and blew and washed for 5 times as well. The following steps were the same as blood specimen.

### Quantitative PCR

5 μl BCV/JCV negative quality control products, specimen and quantitative BCV standard substance I ~ IV were taken and added into PCR tubes. Then they were centrifuged at 2, 000 r/min for 15 s to throwing the liquid on the wall of tube to the bottom of tube. If bubbles flicked the wall of tube, the liquid was centrifuged once more. Then PCR amplification was performed immediately. Detailed procedures are shown in Table-I.

**Table-I:** Conditions and procedures for PCR.

Stage	Temperature	Time	Cycle number
Uracil - DNA Glycocasylase reaction	37 °C	2 min	1
Pre-denaturation	95 °C	3 min	1
Denaturation	94 °C	15 min	40
Annealing, extension and collection of fluorescence signal	60 °C	35 min	
Cooling of equipment	25 °C	1 min	1

### Data analysis

PCR amplified product was detected using gel electrophoresis and meanwhile the detection results was analyzed. Data obtained were analyzed by SPSS 19.0 statistics software. Normal distribution test and homogeneity test of variance were also used. Enumeration data were expressed by percentage.

## RESULTS

### Results of PCR amplification

PCR amplification product obtained from urine and blood was detected using gel electrophoresis. Results are shown in Fig.1.

**Figure:**
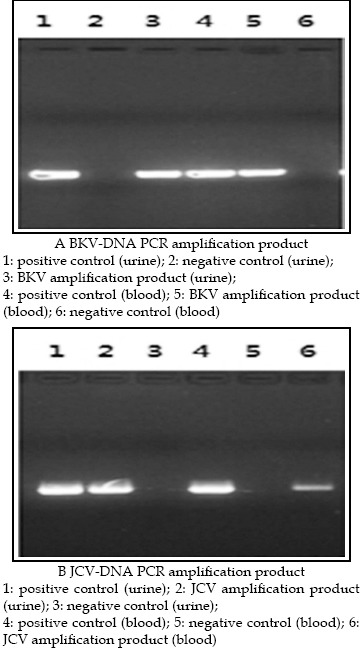
PCR amplification product for BKV and JCV in urine and blood.

### Quantitative detection results of BKV-DNA and JCV-DNA in urine and blood

The lowest detectable limit and linear detection scope for BKV and JCV detection for renal transplantation recipients was 2×10^3^ copies/ml and 5×10^3^ ~ 5×10^8^ copies/ml. We found detection rate and carrying capacity of BKV-DNA was 33.3% and 1.15×10^3^ ~ 6.00×10^11^ copies/ml in urine and 34.8% and 1.3 ×10^3^ ~ 6.06 ×10^5^ copies/ml; detection rate and carrying capacity of JCV-DNA was 30.7% and 1.15×10^3^ ~ 6.00×10^11^ copies/ml in urine and 33.5% and 1.3×10^3^~6.06×10^5^ copies/ml in blood. Median time for occurrence of viruria was 6.1 months and median time for occurrence of viremia was 4.9 months.

### Infection condition of BKV and JCV after renal transplant

**Fig.2:**
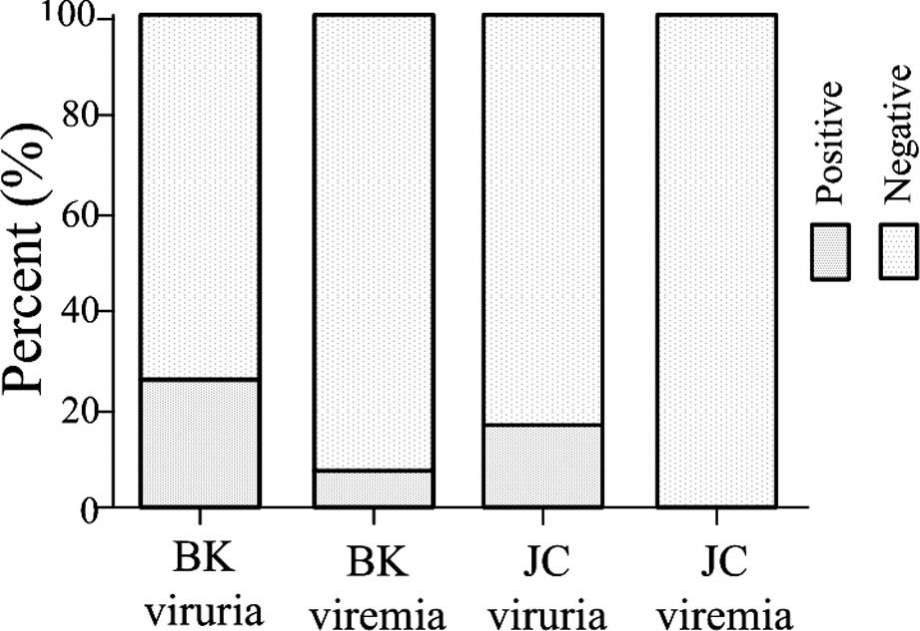
BKV and JCV positive rate after renal transplant.

Urine and blood specimen were collected from three hundred and six renal transplantation recipients in the 5th day, 1st month, 3rd month, 6th month and 12th month after surgery. BKV DNA detection results suggested that, 82 patients had BK viruria, and the positive rate was 26.8%; 25 patients had BK viremia, and the positive rate was 8.2% (Fig.2). JCV DNA detection results suggested that, 51 cases had JC viruria, and the positive rate was 16.7%; and no case developed JC viruria (Fig.2). Besides, no case developed both BKV and JCV infection among 306 cases.

### Sensitivity and specificity of PCR method

Sensitivity of detection of BKV in urine and blood was found to be 98.31% and 99.22% respectively, and its sensitivity was 100% and 99.79% respectively. As to detection of JCV in urine and blood, sensitivity was 97.90% and 98.60% and specificity was both 100%. It proved detecting BKV and LCV with PCR was of high specificity and sensitivity. Detailed data are shown in Table-II.

**Table-II:** Sensitivity and specificity of detecting BCV and JCV with PCR.

Virus	Urine		Blood	

	Sensitivity	Specificity	Sensitivity	Specificity
BKV	98.31%	100%	99.22%	99.79%
JCV	97.90%	100%	98.60%	100%

## DISCUSSION

Polyomavirus belongs to Papovaviridae. Renal transplantation recipients who develop polyomavirus infection are usually found with viruria or obviously abnormity in kidney and urinary system.^9^ 10% ~ 60% renal transplantation recipients infect BKV during anti-rejection treatment, mostly occurring in three months. It has been proved that,^10^ incidence of PVAN forty four weeks after renal transplant is 5%. Patients who are histologically confirmed having PVAN have a possibility of 45% to develop renal allograft dysfunction six month later, if no proper treatment is done.

BKV, one member of polyomavirus family, is able to induce transplant kidney function abnormity or failure; BKV which usually hide away can be reactivated when immune function of host becomes weak.^11^ Thus people who receive immunosuppressive therapy, especially renal transplantation recipients, are more likely to have BKV activation and meanwhile may develop BKV associated nephropathy (BKVAN). BKV mostly hides in kidney; therefore, BKV can be detected out in urine of renal transplantation recipients. Most patients are found with asymptomatic viremia or temporary abnormity in transplant kidney and tissue damage induced by virus may be observed in excised transplant kidney specimen. BKV, considered as the leading cause for PVAN, has 75% sequence homologous with JCV.^12^ JCV can induce progressive multifocal leuko-encephalopathy (PML) and can also replicate in kidney tissue. Application of immunosuppressor in organ transplantation contributes to the activation and replication of polyomavirus; and JCV replication can be detected in 40% over renal transplantation recipients. Incidence of PVAN rises to 15%, if patients infect both JCV and BKV.

In recent years, early diagnosis is thought as the major useful method for PVAN since there are no effective drugs. Diagnostic methods for PVAN are diverse clinically, but people prefer non-invasive detection method, such as virus DNA detection with PCR. Toyoda and Leung et al. has proved that,^13^, ^14^ detecting BKV with quantitative PCR is highly effective. Sensitivity and specificity of detecting polyomavirus with PCR method is 100% and 88% ~ 95%,^15^, ^16^ which is close to the results obtained by the current study. Additionally, it has been reported that, positive rate of Decoy cells in renal transplantation recipients is 29.4% ~40.0%, incidence of viruria is 13.0% ~ 34.7% and incidence of viremia is 5.0%~21.0%. The incidence of viruria mentioned above is consistent with the detection results obtained by this study, but the incidence of viremia is slightly higher. The occurrence of BK viremia may be because that, activated BKV in renal tubular epithelial cell enters into blood circulation through pericanalicular capillary; viremia and viruria are the outcomes of activation of BKV in epithelial cell and peripheral mononuclear cells, and they occur concurrently; virus enters into urine through renal metabolism. Existence of BKV in blood reflects the development process of BKVAN.

### Limitations of the study

The small sample size is one of the limitations of the study. Besides, the detection result of samples between 1×10^3^ and 2×10^3^ may turn out to be false negative as the detection lower limit of kit is 2×10^3^. Most research objects were unwilling to accept puncture biopsy, leading to insufficient clinical data of PVAN diagnosis.

## CONCLUSION

All in all, quantitative and qualitative monitoring of polyomavirus existing in urine and blood can be considered as a useful method for early diagnose and screen BKVAN and PVAN for renal transplantation recipients. Now, researches about polyomavirus are frequently reported, while interaction between PVAN associated with BKV and JCV and immunological rejection of transplant kidney has not been known clearly. We believe that, research with regard to this aspect will be a hot spot and provide new theoretical basis for clinical treatment in the near future.
